# Job satisfaction and perceived workload in the context of personality typology among subway train drivers

**DOI:** 10.3389/fpsyg.2023.1283122

**Published:** 2023-11-28

**Authors:** Dalibor Gottwald, Pavla Lejsková

**Affiliations:** Faculty of Transport Engineering, University of Pardubice, Pardubice, Czechia

**Keywords:** job satisfaction, perceived workload, personality typology, human capital, job performance

## Abstract

Job satisfaction is a highly relevant topic across all sectors of the national economy worldwide. A contented employee significantly enhances a company’s performance compared to a dissatisfied one. Simultaneously, a contented employee increases the human capital value of the company, which has a substantial impact on its overall value. However, employee satisfaction is not a static state; it can be influenced by various factors, one of which is perceived workload resulting from the nature of the job. The aim of this article is to investigate whether there is a demonstrable impact of perceived workload on employee satisfaction. This relationship is examined within the context of subway train drivers in the Czech Republic. In exploring this relationship, we also delve into the psychological factor of whether there is a connection between perceived workload and the satisfaction of subway train drivers based on their personality types according to the MBTI test. These dependencies are assessed through correlation analysis using a comprehensive dataset obtained. In the course of the research, data were collected from the entire basic sample of respondents, namely from 654 subway train drivers. The results confirmed the existing influence of perceived workload on employee satisfaction and, concurrently, the influence of perceived workload on employee satisfaction based on the personality typology determined by the MBTI test. These findings are undoubtedly beneficial for the management of transportation companies, particularly those involved in passenger transportation, specifically in the provision of subway services.

## 1. Introduction

In today’s fast-paced and demanding work environments, understanding the factors that influence job satisfaction and perceived workload has become essential for organizations seeking to optimize employee performance and well-being (Hitka et al., 2019; Sandrin et al., 2019; Chowhan and Pike, 2023; Rubio-Valdehita et al., 2023) . Within the transportation industry, subway train drivers play a crucial role in ensuring the efficient and safe operation of urban transit systems. The job of a subway train driver entails unique challenges, including high levels of responsibility, long working hours, and the need to make split-second decisions while adhering to strict schedules and safety regulations.

While previous research has explored various factors that contribute to job satisfaction and perceived workload among employees, limited attention has been given to understanding the influence of personality typology specifically within the context of subway train drivers. Personality traits can significantly impact individuals’ behaviors, attitudes, and perceptions, and thus understanding the interplay between personality types and job-related outcomes is of paramount importance (Hitka and Stípalová, 2011; Chang et al., 2021; Wang T. et al., 2023).

This research aims to investigate the relationship between personality typology, job satisfaction, and perceived workload among subway train drivers. By examining the diverse personality traits that subway train drivers possess, we seek to shed light on how these traits may influence their perceptions of job satisfaction and perceived workload. The findings of this study have the potential to inform recruitment strategies, employee training programs, and organizational policies, ultimately contributing to the enhancement of work conditions and well-being among subway train drivers.

To accomplish our research objectives, we will employ a comprehensive methodology that combines quantitative and qualitative data collection techniques. We will administer standardized personality assessments to identify distinct personality typologies among subway train drivers. Additionally, we will employ validated measures to assess job satisfaction and perceived workload. By analyzing these data sets, we will explore the associations between personality types, job satisfaction levels, and perceived workload among subway train drivers.

The aim of the study is to examine the significance of the relationship between perceived workload and job satisfaction among subway train drivers. Additionally, the study seeks to determine whether there is a relationship between perceived workload and job satisfaction among subway train drivers based on their personality typology. Simultaneously, the objective is to investigate whether there is a difference in the level of perceived workload and job satisfaction among subway train drivers, specifically in relation to their personality typology. Through this research, we hope to provide valuable insights that empower organizations to foster a positive work environment, enhance employee motivation, and ultimately contribute to the success and well-being of subway train drivers.

### 1.1. Study novelty and contributions

The main contributions of this paper are as follows: (i) The topic of job satisfaction among subway train drivers remains relatively unexplored, with only one existing study that has examined, among other factors, the area of job satisfaction in this profession. Therefore, the contribution of this article becomes evident in its endeavor to expand the scientific knowledge surrounding “job satisfaction” within the scientific community; (ii) By investigating the association between perceived workload and job satisfaction among subway train drivers, the article sheds light on an important but understudied aspect of their work experience. The findings provide valuable insights into the influence of workload perceptions on job satisfaction, helping organizations and policymakers develop strategies to optimize workload management and enhance employee satisfaction. Additionally, the article’s definition of the theoretical concept of employee satisfaction further enriches its contributions in this context; (iii) The article extends the understanding of how personality typology interacts with job satisfaction and perceived workload among subway train drivers. By examining the diverse personality traits present within this occupational group, the study uncovers potential connections between specific personality types and job-related outcomes. This knowledge can inform recruitment processes, training programs, and tailor-made support systems to optimize job satisfaction and well-being among subway train drivers; (iv) The findings of the article hold practical implications for subway transit organizations. By identifying the factors influencing perceived workload and job satisfaction, the study provides valuable insights for designing and implementing targeted interventions. Organizations can utilize these insights to enhance the work environment, improve employee motivation and well-being, and ultimately boost operational efficiency and effectiveness in the subway transportation sector; (v) The article is presented as a case study of subway train drivers, which means it describes the situation in this specific segment. However, the methodologies used to obtain the results can undoubtedly be applied in all other segments as well.

The rest of the paper is structured as follows: Section 2 “Literature review” is a review of the literature. Section 3 “Methodology” elaborates on the methodology. Section 4 “Results” presents the results of the research. Section 5 “Discussion and conclusion” offers the conclusion, and discussion and highlights key avenues for the future research.

## 2. Literature review

Job satisfaction is a critical aspect of organizational behavior and human resource management (Ližbetinová et al., 2020; Lizbetinová et al., 2021; Goetz and Wald, 2022). It refers to an individual’s subjective evaluation of their job and the overall level of contentment they experience in their work environment. Understanding the factors that influence job satisfaction is essential for employers and managers seeking to enhance employee well-being, productivity, and organizational performance.

Several theoretical frameworks have been proposed to explain job satisfaction. One widely recognized theory is the Job Characteristics Model (JCM) developed by Hackman and Oldham (1976). The JCM posits that certain job characteristics, such as skill variety, task identity, autonomy, feedback, and task significance, influence an individual’s psychological states, which, in turn, impact job satisfaction. Another influential theory is the Discrepancy Theory, which suggests that job satisfaction is determined by the discrepancy between an individual’s expectations and their perceived outcomes in the workplace. When expectations align with reality, job satisfaction is higher. Conversely, when there is a significant mismatch, job dissatisfaction may occur.

Various measures have been employed to assess job satisfaction, ranging from global assessments to specific facets of satisfaction. The most widely used instrument is the Job Satisfaction Survey (JSS) developed by Spector (1985). The JSS evaluates satisfaction across different dimensions, including work itself, pay, promotions, supervision, coworkers, and the overall job. Job satisfaction is a complex and multidimensional construct influenced by various factors related to the work environment, compensation, career development, job design, work-life balance, and social support. Understanding the determinants of job satisfaction is essential for organizations aiming to create a positive work environment, retain talented employees, and enhance overall organizational performance. Further research and interventions focusing on these factors can contribute to the development of strategies that promote job satisfaction and employee well-being.

Perceived workload is a crucial factor in understanding employees’ subjective perceptions of the demands and pressures they experience in their work environment. It refers to individuals’ subjective assessment of the quantity and intensity of tasks and responsibilities they perceive in relation to their available resources and capabilities (Kampf et al., 2016; Bartram et al., 2023; Nino et al., 2023) .

Several theoretical frameworks contribute to the understanding of perceived workload. One prominent theory is the Job Demands-Resources (JD-R) model proposed by Demerouti et al. (2001). According to this model, job demands, including workload, can have both negative and positive effects on employee well-being and performance. When the demands exceed an individual’s resources, it can lead to negative outcomes, such as burnout and decreased job satisfaction. Another relevant theory is the Cognitive Load Theory (CLT) introduced by Sweller (1988). CLT posits that individuals have limited cognitive resources and that excessive workload can overwhelm these resources, leading to decreased performance and learning. This theory is particularly applicable in situations where employees are required to process complex information or perform multiple tasks simultaneously.

Perceived workload is a multidimensional construct influenced by various factors related to task characteristics, time pressure, resource availability, job design, workload allocation, and work-life balance (Hasson et al., 2023; Patil et al., 2023). Understanding the determinants of perceived workload is essential for organizations aiming to optimize employees’ well-being, performance, and organizational outcomes. Further research and interventions focusing on these factors can contribute to the development of strategies that effectively manage and mitigate perceived workload, leading to improved employee satisfaction and productivity.

Understanding how individuals perceive and respond to workload demands is essential for optimizing employee well-being and performance. Personality typology, specifically utilizing the Myers-Briggs Type Indicator (MBTI), provides a framework for exploring how different personality traits interact with perceived workload. This literature review examines the relationship between perceived workload and personality typology using the MBTI, focusing on the impact of personality preferences on individuals’ subjective assessment of workload demands.

Assessing perceived workload in the context of MBTI personality typology often involves integrating self-report measures of workload perception with the administration of the MBTI assessment. This combination allows participants to report their subjective workload experiences while capturing their personality preferences based on the MBTI framework.

Current research in Job satisfaction and Perceived workload in transport and communications sector.

Based on a review of scientific articles in the Web of Science database from 2015 to 2023, it is evident that in the field of research on job satisfaction in the transportation and communication sector, several studies have been published. However, only one of them specifically addressed this topic among subway train drivers (see Table 1).

**TABLE 1:** Review of articles from the Web of Science database.

(Chen et al., 2015)	Freight transportation: Occupational injury rates and workplace safety among long-haul truck drivers.
(Buitendach et al., 2016)	Personal transportation: Exploring dimensions of personal well-being among bus drivers in Zimbabwe.
(Sadeghniiat-Haghighi et al., 2016)	Freight transportation: Assessing the prevalence of poor sleep quality among professional long-haul truck drivers with rotating work schedules.
(Huang et al., 2016)	Freight transportation: The relationship between safety climate and job satisfaction among freight truck drivers.
(Zhou et al., 2016)	Personal transportation: The impact of perceived mental workload on work performance among high-speed railway conductors.
(Goncalves and Neves, 2016)	Freight transportation: The influence of burnout syndrome due to chronic occupational stress on turnover among freight truck drivers.
(Prockl et al., 2017)	Freight transportation: the impact of financial and non-financial benefits on job satisfaction among freight truck drivers.
(Jiang et al., 2017)	Freight transportation: The influence of container truck drivers’ satisfaction on the stability of the container trucking industry in China.
(Berrones-Sanz, 2018)	Personal transportation: The effect of working conditions on traffic accident incidents among motorcycle taxi drivers in Mexico City.
(Chen et al., 2018)	Personal transportation: Investigating the interaction between professional psychosocial risks and the influence of metabolic syndrome (METS) on the risk of cardiovascular and cerebrovascular diseases among bus drivers.
(Kwon et al., 2019)	Personal transportation: Surveying factors affecting job satisfaction among bus drivers.
(Hatami et al., 2019)	Freight Transportation: The Impact of Co-Driving on Depression and Occupational Stress among Freight Truck Drivers.
(Kim and Chung, 2019)	Personal transportation: The influence of safety climate (a direct organizational factor) on safety (safe driving) among taxi drivers.
(Wijngaards et al., 2019)	Freight transportation: The impact of diverse job demands on job satisfaction among freight truck drivers.
(Gavilanes-Gavilanes and Moreta-Herrera, 2020)	Freight transportation: The influence of mental health on job satisfaction among heavy cargo drivers in Ecuador.
(Samerei et al., 2020)	Personal Transportation: Researching Factors Influencing Job Satisfaction among Subway Train Conductors in Tehran.
(Chen and Hsu, 2020)	Personal transportation: The impact of emotional exhaustion on work outcomes (including job satisfaction, life satisfaction, organizational commitment, and turnover intention) among bus drivers.
(Shin and Jeong, 2020)	Freight transportation: The influence of negative work situations or work-family conflict on sleep problems and concurrently, whether sleep problems affect job satisfaction among freight truck drivers.
(Thomas et al., 2020)	Freight transportation: The impact of burnout syndrome on the intention to leave the current organization among freight truck drivers.
(Wilhelmsson et al., 2021)	Freight transportation: Research on sustainable working conditions among swine transport drivers in Sweden.
(Joewono et al., 2021)	Personal transportation: Research on factors affecting job satisfaction and productivity among drivers in freight transportation.
(Wygal et al., 2021)	Freight transportation: Research on factors influencing job satisfaction among freight truck drivers in the USA.
(Joseph et al., 2023)	Freight transportation: Investigating causal relationships between categories of risk factors and musculoskeletal disorders among freight truck drivers.
(Zhong et al., 2021)	Personal transportation: The impact of improved lunch on work performance and well-being among bus drivers in Shenzhen, China.
(Silva et al., 2021)	Freight transportation: The influence of perceived work-family conflict on organizational engagement among Portuguese freight truck drivers.
(Delhomme and Gheorghiu, 2021)	Freight transportation: The influence of perceived stress among freight truck drivers on risky driving behaviors.
(Ji-Hyland and Allen, 2020)	Freight transportation: Research on factors contributing to the driver shortage in freight transportation in Ireland.
(Dos Santos, 2021)	Personal transportation: The relationship between stress, job satisfaction, and career decision-making among taxi service drivers in South Korea.
(Wang X. L. et al., 2023)	Personal transportation: the impact of in-car air temperature on cognitive abilities and EEG (standard non-invasive method for functional assessment of central nervous system electrical activity).

Source: Authors.

In the vast majority of the mentioned studies, authors relied on data obtained through primary research methods (questionnaires, interviews) due to the absence of secondary data. Seventeen studies were applied in the field of freight transportation (driver behavior in freight transportation), while twelve studies addressed this issue in the realm of personal transportation, with just one study specifically focused on subway train conductors.

## 3. Methodology

The results presented in the subsequent chapter of the article are based on primary data obtained by the authors through conducting twelve in-depth interviews, followed by a questionnaire survey.

The in-depth interviews were conducted with selected subway train conductors on the following dates: May 19, 2022, May 24, 2022, June 1, 2022, June 13, 2022, and June 14, 2022. The primary research question was: “What topics related to the performance of subway train conductors do you perceive as having an impact on perceived workload?” The total number of respondents was 12, comprising a heterogeneous group (representatives from different age groups, years of service, and subway lines they operate).

The findings from the in-depth interviews, together with the outputs from the literature review, served as the basis for constructing the questionnaire. The questionnaire consisted of three parts. The first part assessed factors related to perceived workload, while the second part evaluated the same factors from the perspective of their influence on overall job satisfaction. In both parts, the factors were rated on a scale of 1–6, where 1 indicated minimal impact, and 6 indicated maximum impact. The third part of the questionnaire involved testing personality typology using the Myers-Briggs Type Indicator. The Myers-Briggs Type Indicator (MBTI) is a widely used personality assessment tool that categorizes individuals into specific personality types. It is based on the theories of Carl Jung and measures four dichotomous dimensions of personality: Extraversion (E) – Introversion (I): Determines whether individuals are energized by external stimulation and social interaction (extraversion) or by internal thoughts and solitude (introversion). Sensing (S) – Intuition (N): Reflects how individuals perceive information. Sensing individuals focus on concrete details and rely on their senses, while intuitive individuals are more inclined to interpret patterns and rely on their instincts. Thinking (T) – Feeling (F): Describes how individuals make decisions. Thinking individuals tend to prioritize logic and objective analysis, whereas feeling individuals emphasize personal values and the impact on others. Judging (J) – Perceiving (P): Represents how individuals approach the outside world. Judging individuals prefer structure, planning, and order, while perceiving individuals are more flexible, adaptable, and open-ended. By combining these dimensions, the MBTI generates 16 distinct personality types, such as ISTJ (Introverted, Sensing, Thinking, Judging) or ENFP (Extraverted, Intuitive, Feeling, Perceiving). The MBTI provides insights into individuals’ preferences, behaviors, and potential career choices, and it has been used in various fields, including psychology, education, and organizational development.

The questionnaire survey took place from September 19, 2022, to October 27, 2022. The survey included all subway train conductors of the transport company, totaling 654 subway train conductors. After data cleansing, a total of 589 responses from respondents were retained for subsequent testing. 65 responses were excluded due to unwillingness to complete the questionnaire or due to incorrectly filled out questionnaires.

Normality of the data distribution was tested in the obtained dataset. Subsequently, hypotheses were tested using a method for examining the relationships between variables.

### 3.1. Normality test

To assess the normality of the data distribution, a normality test was conducted using the Kolmogorov-Smirnov method. This test is a non-parametric test that compares the empirical cumulative distribution function (ECDF) of the sample data to the cumulative distribution function (CDF) of a theoretical normal distribution. The null hypothesis (H0) assumes that the data follows a normal distribution, while the alternative hypothesis (H1) suggests that the data deviates significantly from a normal distribution.

The study involved a sample of 589 participants from subway train drivers (the entire population after cleaning the data set). Data was collected using questionnaire. The participants were instructed to fill out a questionnaire. The collected data consisted of values 1 to 6.

Normality test procedure by Kolmogorov-Smirnov method:

(a) Descriptive Statistics: Descriptive statistics such as mean, standard deviation, skewness, and kurtosis were calculated to examine the distributional characteristics of the data.
(b) Hypotheses Formulation: The null hypothesis (H0) assumed that the data followed a normal distribution, while the alternative hypothesis (HA) stated that the data deviated significantly from normality.
(c) Kolmogorov-Smirnov Test: The Kolmogorov-Smirnov test was performed to assess the normality assumption. The test statistic, D, was calculated as the maximum absolute difference between the ECDF of the sample data and the CDF of the theoretical normal distribution.
(d) Significance Level: The significance level (α) was set at 0.05 to determine the critical value for rejecting the null hypothesis. If the *p*-value associated with the test statistic was less than α, the null hypothesis was rejected, indicating non-normality of the data.
(e) Interpretation of Results: The test results were interpreted based on the *p*-value. If the *p*-value was greater than α, we failed to reject the null hypothesis, suggesting that the data followed a normal distribution. Conversely, if the *p*-value was less than α, we rejected the null hypothesis in favor of the alternative hypothesis, indicating a significant deviation from normality.

### 3.2. Formulated hypotheses

H0_1_: Perceived workload has no effect on job satisfaction among subway train conductors.

HA_1_: Perceived Workload does have an effect on job satisfaction among subway train conductors.

H0_2_: Perceived Workload has no effect on job satisfaction among subway train conductors with respect to their personality typology.

HA_2_: Perceived Workload does have an effect on job satisfaction among subway train conductors with respect to their personality typology.

### 3.3. Testing hypotheses

The Spearman correlation coefficient is a statistical measure used to determine the strength and direction of the relationship between two variables, without assuming any specific distributional form. This article presents a comprehensive methodology for calculating the Spearman correlation coefficient in research studies. The methodology covers data preparation, calculation procedure, and interpretation of results. By following this methodology, researchers can effectively analyze the relationship between variables and draw reliable conclusions.

Calculation of Spearman correlation coefficient:

(a) Rank Assignment: Assign ranks to the values of each variable separately. If ties occur, assign the average rank to tied observations.
(b) Calculate Differences: Calculate the difference between the ranks of each pair of observations for both variables.
(c) Calculate Squared Differences: Square the differences obtained in the previous step.
(d) Sum Squared Differences: Sum the squared differences.
(e) Calculate the Spearman Correlation Coefficient with using the formula:

(1) $$r_{s}=1-\frac{6\,\sum_{i}d_{i}^{2}}{n\,\left(n^{2}-1\right)}$$

where:

*n* … number of data points of the two variables

*d_i_* … difference in ranks of the *i* element

The Spearman Coefficient can take a value between +1 to −1 where,

*r_s_* value of +1 means a perfect association of rank

*r_s_* value of 0 means no association of ranks

*r_s_* value of −1 means a perfect negative association between ranks.

## 4. Results

Testing Data Normality Using the Kolmogorov-Smirnov Method for Scientific Articles.

– Sample size: 11 780 “(A total of 589 subway train drivers assessed a total of 20 questions, with 10 questions related to perceived workload and 10 questions related to job satisfaction).”
– Test statistic D: 0.1677
– *P*-value: 0

**FIGURE 1:**
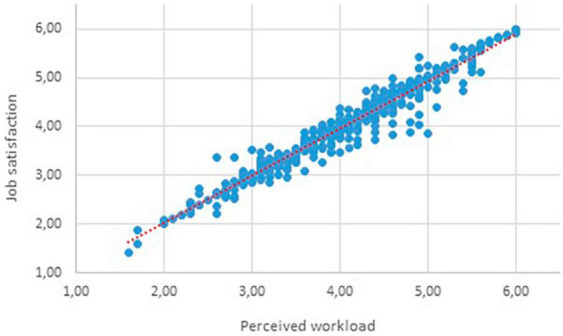
Graphical representation of the application of the Spearman correlation coefficient. Source: Authors.

Results:

– The test statistic D equals 0.1677, which is not in the 95% region of acceptance: [−:0.008583]
– *P*-value (0) < α (0.05), we reject the H0.
– It is assumed that the data distribution is not normal. The difference between the data sample and the normal distribution is big enough to be statistically significant. Based on these results, it is possible to proceed with testing the formulated hypotheses using the Spearman Correlation Coefficient. Testing hypotheses using the Spearman Correlation Coefficient:
– The impact of perceived workload on the job satisfaction of subway train drivers (H_1_).
– Based on the results presented in Table 2, it is possible to reject H0_1_ and accept HA_1_. It can therefore be concluded that there is an influence of perceived workload on the job satisfaction of subway train drivers, and this influence is indeed highly significant. This trend can also be observed in the function plotted in Figure 1.
– The impact of workload on the job satisfaction of subway train drivers considering their personality typology (H_2_).

**TABLE 2:** The result of testing hypotheses using the Spearman correlation coefficient.

Number of XY Pairs	589
Spearman *r*	0.9760
*P*-value (two-tailed)	*P* < 0.0001
*P*-value summary^1^	***
Is the correlation significant? (α = 0.05)	YES

^1^Significance level of the dependency according to the *P*-value. Source: Authors.

Based on the results presented in Table 3, it is possible to reject H0_2_ and accept HA_2_. Therefore, it can be concluded that there is an influence of perceived workload on the job satisfaction of subway train drivers, considering their personality typology. This influence is indeed highly significant (the correlation coefficient value ranges from 0.9333 to 1.000).

**TABLE 3:** The results of testing hypotheses using the Spearman correlation coefficient with respect to MBTI typology.

Osobnostní typologie (dle MBTI)^1^	Number of Pairs	Spearman r	*P*-value (two-tailed)	*P*-value summary^3^	Is the correlation significant?
ENFJ	9	0.9333	0.0007	***	YES
ENFP	5	1.000	0.0167	*	YES
ENTJ	14	0.9680	*P* < 0.0001	***	YES
ENTP^2^	1	—	—	—	—
ESFJ	53	0.9401	*P* < 0.0001	***	YES
ESFP	10	0.9787	*P* < 0.0001	***	YES
ESTJ	106	0.9792	*P* < 0.0001	***	YES
ESTP	5	1.000	*P* < 0.0001	***	YES
INFJ	15	0.9803	*P* < 0.0001	***	YES
INFP	8	0.9819	*P* < 0.0001	***	YES
INTJ	13	0.9972	*P* < 0.0001	***	YES
INTP^2^	3	–	–	–	–
ISFJ	107	0.9772	*P* < 0.0001	***	YES
ISFP	19	0.9705	*P* < 0.0001	***	YES
ISTJ	201	0.9793	*P* < 0.0001	***	YES
ISTP	20	0.9857	*P* < 0.0001	***	YES

Source: Authors.

^1^ ENFJ, Extraversion, Intuition, Feeling, Judgment; ENFP, Extraversion, Intuition, Feeling, Perceiving; ENTJ, Extraversion, Intuition, Thinking, Judgment; ENTP, Extraversion, Intuition, Thinking, Perceiving; ESFJ, Extraversion, Sensing, Feeling, Judgment; ESFP, Extraversion, Sensing, Feeling, Perceiving; ESTJ, Extraversion, Sensing, Thinking, Judgment; ESTP, Extraversion, Sensing, Thinking, Perceiving; INFJ, Introversion, Intuition, Feeling, Judgment; INFP, Introversion, Intuition, Feeling, Perceiving; INTJ, Introversion, Intuition, Thinking, Judgment; INTP, Introversion, Intuition, Thinking, Perceiving; ISFJ, Introversion, Sensing, Feeling, Judgment; ISFP, Introversion, Sensing, Feeling, Perceiving; ISTJ, Introversion, Sensing, Thinking, Judgment; ISTP, Introversion, Sensing, Thinking, Perceiving.

^2^ Due to the limited representation of subway train operators among the personality types ENTP and INTP, validity testing of these hypotheses was not conducted for these two typologies.

^3^ Significance level of the dependency according to the P-value.

**FIGURE 2:**
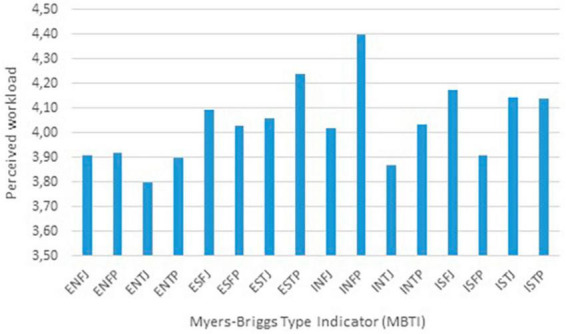
Differences in the assessment of selected factors from the perspective of perceived workload. Source: Authors.

Figure 2 illustrates the differences in the assessment of selected ten factors from the perspective of perceived workload among subway train drivers. Based on the demonstrated relationship between perceived workload and job satisfaction, taking into account the personality typology of subway train drivers, it can be concluded that the selected typologies [Extraversion, Intuition, Thinking, Perceiving (ENTP), Introversion, Intuition, Thinking, Judgment (INTJ), Introversion, Sensing, Feeling, Perceiving (ISFP), Extraversion, Intuition, Feeling, Judgment (ENFJ), and ENFP] perceive workload less than other typologies (INFP, ESTP, and ISFJ).

## 5. Discussion and conclusion

**FIGURE 3:**
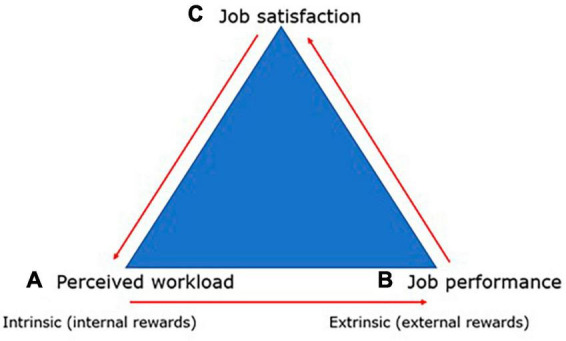
Triangle of the relationship between perceived workload, job satisfaction, and job performance. Source: Authors.

Based on the findings, it is possible to confirm the significant impact of perceived workload on job satisfaction among subway train drivers, even in the context of personality typology. These results clearly contribute to understanding the process of building the status of a satisfied employee. In the context of this discovery, it is possible to define a general theoretical concept of employee satisfaction that will be applicable to any job position (see Figure 3).

Employee satisfaction is a combination of both intrinsic and extrinsic contentment with work. Intrinsic job satisfaction (depicted as “a” in the triangle on Figure 3) is directly linked to the type of work the employee performs. The type of work can also be defined through factors that impact perceived workload. Each type of work will have different factors–in our case, these were factors characterizing perceived workload among subway train drivers (Perceived feelings of fatigue and drowsiness; Pressure due to a sense of responsibility; Pressure from passenger behavior on platforms; Long and irregular shifts; Lack of natural light; Underground environment; Noise; Location of facilities; Quality of social amenities; Air quality). Perceived workload in relation to job type then influences the work performance of the employee (depicted as “b” in the triangle on Figure 3). The greater the perceived workload, the lower the work performance. This relationship becomes more pronounced in the long term. Perceived workload reflected in work performance directly affects employee job satisfaction (depicted as “c” in the triangle on Figure 3). Each job type is associated with factors that induce or cause workload. However, if the influence of these factors is minimized, this fact positively affects higher employee satisfaction. This elucidates why the “process” of enhancing employee job satisfaction is a never-ending journey.

In our research, we focused on the relationship between perceived workload and job satisfaction among subway train drivers. However, it’s important to recognize that employee satisfaction is a complex phenomenon influenced by a wide range of factors. Therefore, it’s crucial to discuss other aspects that contribute to overall job contentment.

One potentially important aspect to consider is organizational culture. Different organizational cultures can have various impacts on employee satisfaction levels. For instance, support for team spirit and emphasis on creating a positive work environment can significantly affect employee satisfaction. Conversely, an organizational culture that overly stresses performance and competitiveness might cause stress and reduce job satisfaction (Al-Shammari and Al-Am, 2018; Khan et al., 2023).

Another potentially significant aspect is leadership, i.e., leadership style and managerial practices. Quality of leadership and managers’ ability to communicate with subordinates can play a key role in fostering a positive work environment. Inspirational and supportive leadership can improve employee motivation and engagement, which ultimately positively affects job satisfaction (Guevara et al., 2019; Ahmad et al., 2021).

Furthermore, personality factors should not be overlooked. Different personality types may react differently to the work environment and job demands. Some personalities might be more resilient to stress and pressure, while others might require specific conditions and support to achieve optimal job satisfaction (Steel et al., 2019; Kang and Malvaso, 2023). The influence of personality factors, specifically the influence of employee personality typology on perceived workload and job satisfaction, was also addressed in our research. For all personality types examined, a significant influence of perceived workload on job satisfaction among subway train drivers was confirmed. Through a more detailed analysis, differences in perceived workload among individual subway train operator personality typologies were also examined. Based on the results, it can be concluded that certain personality types (ENTP, INTJ, ISFP, ENFJ, and ENFP) perceive workload less than other typologies (INFP, ESTP, and ISFJ). These findings are certainly beneficial for management representatives, especially those in transportation companies operating subways, who can take these findings into account when selecting new subway train drivers. This conclusion can be generalized beyond this segment as well. However, for different segments, similar testing would need to be conducted due to the likelihood that each type of work will be more or less suitable for a specific personality type, at least in terms of perceived workload. In this context, there is room for further research with the ambition of discovering in which industries specific employee personality typologies are associated with greater or lesser resilience to perceived workload, which ultimately affects job satisfaction.

With reference to the results of our research, it is also possible to delineate an area for further related research in the field of personality typology in the context of job satisfaction and perceived workload. Specifically, it would be advisable in further research to focus on other professions and to compare job satisfaction and perceived workload within the context of personality typology across different states.

The insights provided can serve as valuable guidance for management representatives in enhancing employee satisfaction and optimizing workplace conditions within subway-operating companies. Understanding the nuanced interplay between perceived workload, personality typology, and job satisfaction can aid in the development of effective HR strategies and policies that promote employee well-being and performance. This integration of research findings into practical applications can contribute to fostering a more conducive and fulfilling work environment, ultimately leading to improved organizational productivity and employee retention.

Although this research sheds light on the relationship between perceived workload and job satisfaction among subway train drivers, there are certain limitations that need to be acknowledged. Firstly, the study focused solely on the subway train driver profession, potentially limiting the generalizability of the findings to other job positions. Other professions may have unique dynamics and factors influencing job satisfaction that were not considered in this study. Secondly, the study primarily examined the influence of perceived workload on job satisfaction, without delving extensively into other potential factors that could contribute to overall job contentment. As a result, the comprehensive understanding of the intricate nature of employee satisfaction might not have been fully captured. Furthermore, the research heavily relied on self-report measures and subjective assessments, which might have introduced a certain degree of bias or inaccuracy. Objective measures or a more diverse set of data collection methods could provide a more comprehensive and nuanced perspective on the relationship between perceived workload, personality typology, and job satisfaction. Lastly, the research was conducted within a specific cultural and organizational context, which might limit the generalizability of the findings to different cultural or geographical settings. Cultural variations and organizational structures can significantly impact the dynamics of perceived workload and job satisfaction, and these variations were not extensively explored in this study. Despite these limitations, the findings of this research provide a valuable foundation for understanding the intricate relationship between perceived workload, personality typology, and job satisfaction, offering a basis for further exploration and refinement in the field.

## 6. Conclusion

This article successfully contributes to filling gaps in the area of perceived workload and job satisfaction research, specifically among subway train drivers. A general theoretical concept of employee satisfaction has been established. Moreover, a connection has been made between perceived workload and job satisfaction in the context of personality typology. This insight can be seen as a practical contribution, especially for management representatives in subway-operating companies. Additionally, it can be noted that the methodology used is applicable across all segments, not just among subway train drivers.

## Data availability statement

The original contributions presented in this study are included in the article/supplementary material, further inquiries can be directed to this corresponding author.

## Ethics statement

Ethical approval was not required for the study involving humans in accordance with the local legislation and institutional requirements. The studies were conducted in accordance with the local legislation and institutional requirements. The participants provided their written informed consent to participate in this study. Written informed consent was obtained from the individual(s) for the publication of any potentially identifiable images or data included in this article.

## Author contributions

DG: Conceptualization, Methodology, Supervision, Validation, Writing — original draft. PL: Data curation, Methodology, Resources, Writing — review and editing.
